# Gelatin hydrogel/contact lens composites as rutin delivery systems for promoting corneal wound healing

**DOI:** 10.1080/10717544.2021.1979126

**Published:** 2021-09-25

**Authors:** Lianghui Zhao, Xia Qi, Tao Cai, Zheng Fan, Hongwei Wang, Xianli Du

**Affiliations:** a Qingdao Eye Hospital of Shandong First Medical University, Qingdao, Shandong, China; b State Key Laboratory Cultivation Base, Shandong Provincial Key Laboratory of Ophthalmology, Shandong Eye Institute, Shandong First Medical University & Shandong Academy of Medical Sciences, Qingdao, Shandong, China

**Keywords:** Gelatin hydrogel/contact lens composites, free radical polymerization, CMC/NHS crosslinking, corneal wound healing, rutin, proteomics

## Abstract

Corneal wound healing is a highly regulated biological process that is of importance for reducing the risk of blinding corneal infections and inflammations. Traditional eye drop was the main approach for promoting corneal wound healing. However, its low bioavailability required a high therapeutic concentration, which can lead to ocular or even systemic side effects. To develop a safe and effective method for treating corneal injury, we fabricated rutin-encapsulated gelatin hydrogel/contact lens composites by dual crosslinking reactions including *in situ* free radical polymerization and carboxymethyl cellulose/*N*-hydroxysulfosuccinimide crosslinking. *In vitro* drug release results evidenced that rutin in the composites could be sustainedly released for up to 14 days. In addition, biocompatibility assay indicated nontoxicity of the composites. Finally, the effect of rutin-encapsulated composites on the healing of the corneal injury in rabbits was investigated. The injury was basically cured in corneas using rutin-encapsulated composites (healing rate, 98.3% ± 0.7%) at 48 h post-operation, while the damage was still present in corneas using the composite (healing rate, 87.0% ± 4.5%). Further proteomics analysis revealed that corneal wound healing may be promoted by the ERK/MAPK and PI3K/AKT signal pathways. These results inform a potential intervention strategy to facilitate corneal wound healing in humans.

## 1. Introduction

The cornea, an important biological barrier of the eye, can resist the invasion of chemical substances and microorganisms to a certain extent and protect eye health (Ziaei et al., 2018). Cornea injury may cause serious complications, such as infection, ulcer or even perforation, which seriously threatens vision and affects patients’ quality of life. Corneal wound healing is a highly regulated biological process that is of utmost importance for reducing the risk of potentially blinding corneal infections and perforation (Nishida et al., 2015; Zhu et al., 2019). At present, traditional eye drops were the main approach for the treatment of the corneal injury. Due to the low bioavailability, a high therapeutic concentration is often used to treat ophthalmic disease, which is likely to cause ocular and systemic side effects. In addition, the preservatives in traditional eye drops can damage the health of the ocular surface. Thus, it is valuable to develop an alternative method for safe and effective therapy of corneal injury (Liu et al., 2019; Ljubimov & Saghizadeh, 2015).

The contact lens has been a ubiquitous medical device for treating ophthalmic diseases and correcting refractive errors (Ciolino et al., 2014; Huang et al., 2016; Kim et al., 2014; Qin et al., 2017; Ross et al., 2019). They can act as a bandage lens to protect the epithelium from the injury of eyelid closure or other factors, and can also be a drug-delivery system to improve the bioavailability of drugs for facilitating wound healing. Nevertheless, long-term wearing and non-compliant operations of contact lenses could lead to inflammation, infection, and other ocular complications. Natural biological polymer gelatin is the collagen hydrolysate of the extracellular matrix. The gelatin hydrogels can be prepared by multiple crosslinking approaches such as physical, chemical and enzyme crosslinking reactions. They exhibited good biocompatibility, optical properties and non-immunogenicity, and are widely used in the field of drug delivery and regenerative medicine (Annamalai et al., 2018; Dhand et al., 2017; Diba et al., 2021; Ding et al., 2021; Ping et al., 2021; Xuan et al., 2020; Zhang et al., 2020). Nowadays, gelatin hydrogels have been considered as an emerging biomaterial for the restoration and regeneration of the damaged cornea tissue (Cui et al., 2021; Khosravimelal et al., 2021). However, the poor mechanical strength of the gelatin hydrogels impeded their applications as medical devices such as contact lenses for the treatment of ophthalmic diseases. Hence, it is expected to develop the gelatin hydrogel/contact lens composites to combine the complementary advantages of traditional contact lenses and gelatin hydrogel, and provide a new carrier for promoting wound healing (Assmann et al., 2017; Liu et al., 2018; Nie et al., 2018; Wang et al., 2019).

Quercetin, a member of the flavonoid family, is an important second metabolite that is widely distributed in vegetables, fruits, grains, tea, red wine, and so on. The flavone aglycone exhibits various biological activities, including antioxidant, anti-inflammatory, anti-fibrosis, immunomodulatory, anti-cancer, vascular protection, and neuroprotection functions (Quideau et al., 2011; Veitch, 2013; Lu et al., 2011). It has been used for the treatment of keratoconus, Graves ophthalmopathy, conjunctivitis, cataract, dry eye disease, retinopathy, and other ophthalmological diseases (Zhao et al., 2021). Rutin, the glycoside form of quercetin, has also been demonstrated efficacy for the prevention and treatment of inflammation, cataract, retinal neurovascular disease, and other ophthalmic diseases (Ola et al., 2015; Sasikala et al., 2013). However, the poor solubility of rutin in aqueous media leads to poor bioavailability after administration, which restricts the direct clinical applications. Several strategies, including cyclodextrins complexation, nanocarriers, and hydrogel carriers, have been proposed in recent years for enhancing the bioavailability of rutin (Júlio et al., 2019; Lee et al., 2018; Paczkowska et al., 2020). Among them, hydrogel offers a convenient and readily available approach.

**Figure 1.**
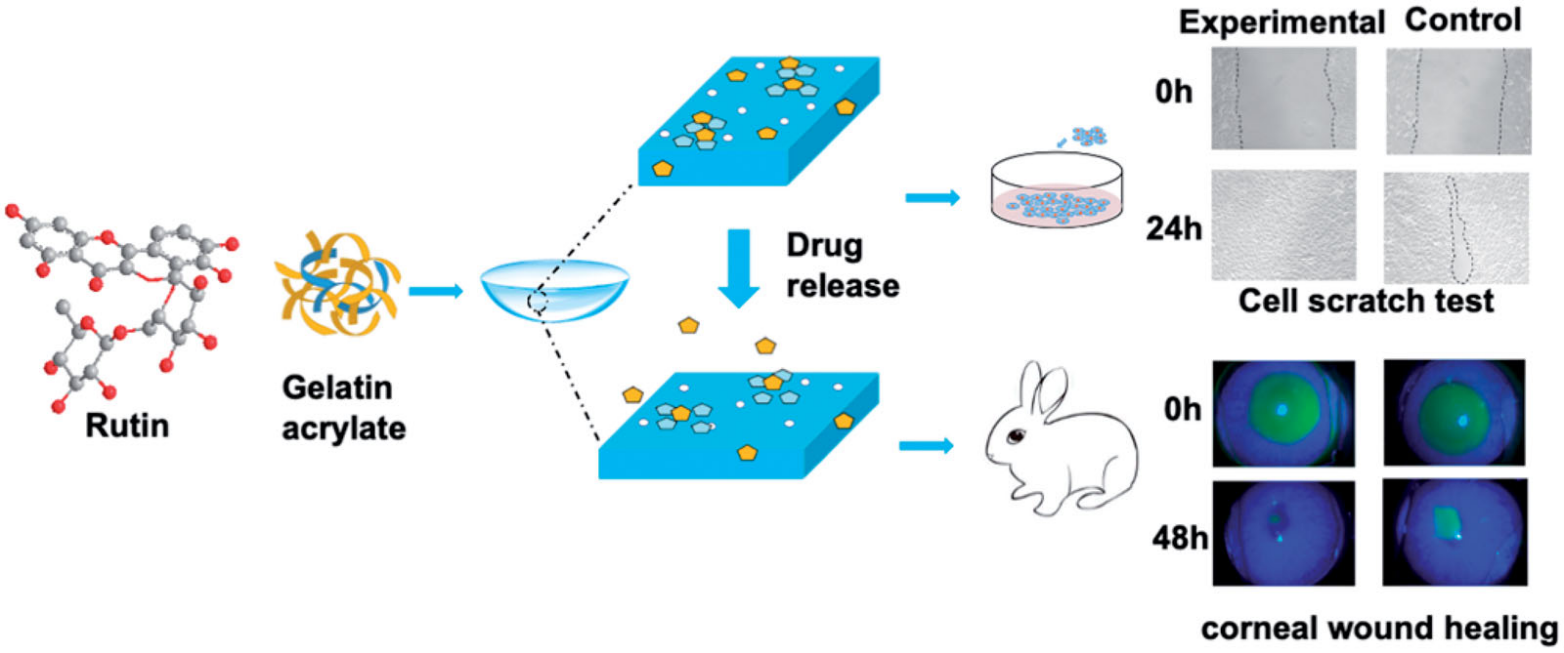
Scheme for the construction of gelatin hydrogel/contact lens composites as rutin delivery systems to promote corneal wound healing.

Herein, we reported the fabrication of rutin-encapsulated gelatin hydrogel/contact lens composites by *in situ* free-radical polymerization and carboxymethyl cellulose/N-hydroxysulfosuccinimide (CMC/NHS) crosslinking reactions of a mixture containing ethylene glycol dimethylacrylate (EGDMA), 2-hydroxylethyl methacrylate (HEMA), methacrylic acid (MAA), gelatin, rutin, CMC and NHS (Figure 1). For comparison, another composite was prepared using a similar method with rutin-encapsulated gelatin hydrogel/contact lens composites except for the absence of CMC and NHS in the system. Two types of the resulting composites were assessed and compared in terms of their physical characterizations, *in vitro* rutin release and *in vitro* biocompatibility. The rutin-encapsulated gelatin hydrogel/contact lens composites were then applied to promote corneal wound healing using the corneal injury model of rabbits, and the corresponding molecular mechanism was further investigated through proteomic analysis.

## 2. Materials and methods

### 2.1. Materials

HEMA, MAA, gelatin, acrylic anhydride, and rutin were obtained from the Macklin Company (Shanghai, China). EGDMA, 2,2′-azobis (2-methylpropionitrile) (AIBN), CMC, and NHS were bought from the Aladdin Company (Shanghai, China).

### 2.2. Preparation of rutin-encapsulated gelatin hydrogel/contact lens composites

Firstly, gelatin (5.0 g) and deionized water (45.0 mL) were thoroughly stirred at 50 °C until complete dissolution. After the addition of acrylic anhydride (5.0 mL), the mixture was reacted at 50 °C for 24 h, subsequently dialyzed for 6 days, and freeze-dried for 5 days to prepare acrylated gelatin. Various amounts of rutin (0%, 0.3%, 0.6%, 1% and 1.3 wt%), HEMA/MAA (70/30, 70 wt%), EGDMA (0.45 wt%), acrylated gelatin (33.3 wt%), CMC (0.15 wt%), NHS (0.03 wt%) and AIBN (0.15 wt%) were mixed into the deionized water (Liu et al., 2016). The mixture was sonicated for 5 min, passed through a 0.22‐μm filter, and transferred into the contact lens molds. The molds were placed in a water bath at 65 °C for 30 h and then boiled for 5 min to remove the unreacted monomers, thus affording contact lenses (CL-0 – CL-4). In addition, four composites (CL-0′– CL-4′) were prepared by a similar method except for the absence of CMC and NHS. Furthermore, conventional contact lenses (CL) were also prepared by the free radical polymerization of HEMA/MAA (70/30, 70 wt%), EGDMA (0.45 wt%), and AIBN (0.15 wt%).

### 2.3. Physical characterizations

The transparency of the rutin-encapsulated gelatin hydrogel/contact lenses was assessed by placing the lenses on a white paper bearing a black letter ‘A’. The optical transmittance of contact lenses was measured by an ultraviolet-visible spectrophotometer (SpectraMax M2, Molecular Devices, MD, USA) at 50 nm intervals in the wavelength range of 250–800 nm. The swelling performance was evaluated by immersing the dried contact lenses in deionized water. The swelling ratio was calculated with the following equation: swelling ratio (%) = (*Wt*−*Wd*)/*Wd*, where *Wt* and *Wd* represent the wet weight and dry weight, respectively. Thermogravimetric analysis (TGA) was performed using a TA SC-TGA Q600 (USA), and Fourier transform infrared spectrum analysis (FTIR) was measured by Bruker Tensor II (Germany). Energy-dispersive X-ray spectroscopy (EDS) mapping was recorded by Merlin Compact (ZEISS, Germany). X-ray photoelectron spectroscopy (XPS) was collected using ESCALAB 250Xi XPS spectrometer (Thermo Fisher Scientific, USA).

### 2.4. *In vitro* release study

The rutin-encapsulated gelatin hydrogel/contact lens composites were immersed in deionized water (4.0 mL). At pre-established time intervals, 1.0 mL of the release medium was taken out and replaced with an equal amount of fresh deionized water. HPLC (Agilent, USA) was used to determine the rutin content for plotting the cumulative release curve of rutin with time by GraphPad Prism 9. The experiment was repeated three times.

### 2.5. Cell assays

#### 2.5.1. Cytotoxicity test

Human corneal epithelial cells (HCECs) were provided by Professor Choun-Ki Joo from the School of Medicine, Catholic University of Korea (Seoul, Korea). Rutin-encapsulated hydrogel/contact lens composites (2.5 mg, 5.0 mg and 10.0 mg) were dipped into 10.0 mL of DMEM/F12 (Sigma-Aldrich) supplemented with 10% (v/v) fetal bovine serum (FBS, Sigma-Aldrich) and 1% (v/v) antibiotic/antimycotics (Sigma-Aldrich) in 37 °C and 5% CO_2_ culture environments for 24 h to obtain the extracts of the composites with concentrations of 1:4, 1:2 and 1:1, respectively (Li et al., 2020). The cytotoxicity of the composites was evaluated by investigating the effects of extracts on the proliferation of HCECs with a cell counting kit-8 (CCK-8) assay. In brief, the HCECs were seeded into a 96-well plate at a density of 2000 cells/well and cultured in standard culture mediums (DMEM/F12, 10% FBS, 1% antibiotic/antimycotics) for 1 day at 37 °C and 5% CO_2_. In the next day, standard culture media in experimental and control groups were replaced with the prepared contact lenses extracts and standard culture medium, respectively. The cell proliferation abilities were evaluated with the CCK-8 assay kit (Dojindo Laboratories Kumamoto, Japan) after incubation for 1, 3, 5, and 7 days at 37 °C and 5% CO_2_.

#### 2.5.2. Cell scratch test

The HCECs were inoculated into a 12-well plate at a density of 1 × 10^5^ cells/well. At 90% confluence, wounds were created with a 1-mL pipette tip in the middle of the monolayer. The damaged cells were removed by washing with PBS, and the remaining cells were continuously cultured by the addition of the standard medium and the extract, respectively. Photographs were captured to record the wound healing at a predetermined time interval. The experiment was repeated for three iterations to ensure the authenticity and reliability of the results.

### 2.6. Animal studies

The animal experiments were approved by the Ethics Committee of the Shandong Eye Institute (China). Besides, all experiments were conducted in accordance with the Association for Research in Vision and Ophthalmology Statement’s guidelines for the use of animals in ophthalmic and visual research. Male New Zealand white rabbits (2.5–3.0 kg) were purchased from the Xilingjiao Animal Breeding Center (Jinan, China). Before the experiment, all rabbits were kept in an animal room with suitable temperatures and sufficient food for one week to ensure their health and exclude the external influencing factors that may influence the experiment.

#### 2.6.1. *In vivo* biocompatibility

Rutin-encapsulated gelatin hydrogel/contact lens composites were worn in the eyes of normal rabbits for 6 h per day on 4 consecutive days. The corneas were observed by a slit lamp microscope and photographed every day (*n* = 3).

#### 2.6.2. Corneal wound healing in rabbits

CL-0 and CL-2 were selected to assess the effect of rutin-encapsulated gelatin hydrogel/contact lens composites on corneal wound healing using the corneal injury model of rabbits. The right and left eyes of the rabbits were selected as the control (CL-0) and experimental groups (CL-2), respectively. After anesthesia, a 9-mm trephine was adopted for imprinting the surgical area in the corneal center, and the corneal epithelium in the area was resected with an electric epithelial scraper. Levofloxacin eye drops were given to both eyes after surgery. At predetermined time intervals (0, 12, 24, 36, and 48 h), the corneal epithelium of rabbits in two groups was stained by fluorescein sodium staining and photographed by slit-lamp microscope (*n* = 6). The rabbits were euthanized (*n* = 3) at 48 h post-operation, and the corneas were collected for hematoxylin-eosin staining and proteomic analysis.

#### 2.6.3. Proteomic analysis

The corneas were collected from the euthanized rabbits to extract the corneal proteins and investigate the total proteomics through LC-MS/MS analysis (*n* = 3). The extracted proteins were enzymolysis with trypsin, and the protein-peptide fragments were labeled with a TMT-10PLEX reagent. Then each sample was divided into 12 groups by the C18 column (Thermo Fisher Scientific, America). Quantitative proteomics was applied to identify the differentially expressed proteins in combination with gene ontology (GO) enrichment analysis, Kyoto encyclopedia of genes and genomes (KEGG) enrichment analysis, domain enrichment, and protein interaction analysis.

EASY-nLC 1200 nano liquid chromatography system (Thermo Fisher Scientific, USA) coupled with an Orbitrap Fusion Lumos mass spectrometer (Thermo Fisher Scientific, USA) were adopted for LC-MS/MS analysis. Mobile phase A (H_2_O/formic acid, 99.9: 0.1, v/v) and B (acetonitrile/formic acid, 99.9: 0.1, v/v) were used for the UPLC separation. The peptides mixture was separated by a 15 cm × 150 μm C18 column at a flow rate of ∼600 nL/min. The gradient elution was carried out as in Tables S1–S6 (supporting information). All the LC-MS/MS raw data were searched with Proteome Discoverer (version 2.4) against a SEQUEST database. The mass tolerances were 20 ppm for initial precursor ions and 0.05 Da for-fragment ions. Two missed cleavages were permitted for trypsin restriction. The cutoff false discovery rate for all peptide identifications was controlled below 1%.

For functional analysis, the differentially-expressed proteins were classified using the terms from KEGG and GO databases. The KEGG and GO enrichment analysis was performed by means of the hypergeometric distribution test and Fisher’s exact test, respectively. A *p*-value of less than .05 was considered statistically significant.

### 2.7. Statistical analysis

Statistical analysis was performed using Statistical Package for Social Sciences software (version 24.0). The normality of all data was checked with the Kolmogorov–Smirnov test. Independent *t*-test and Kruskal-Wallis test were used to compare the rate of wound healing and the absorbance of CCK-8, respectively, between different groups. All of the data were obtained from at least three independent experiments. The descriptive statistics were presented as the mean ± standard deviation (SD). *P* < .05 was considered to be a statistically significant difference.

## 3. Results

### 3.1. Synthesis and characterizations

**Figure 2.**
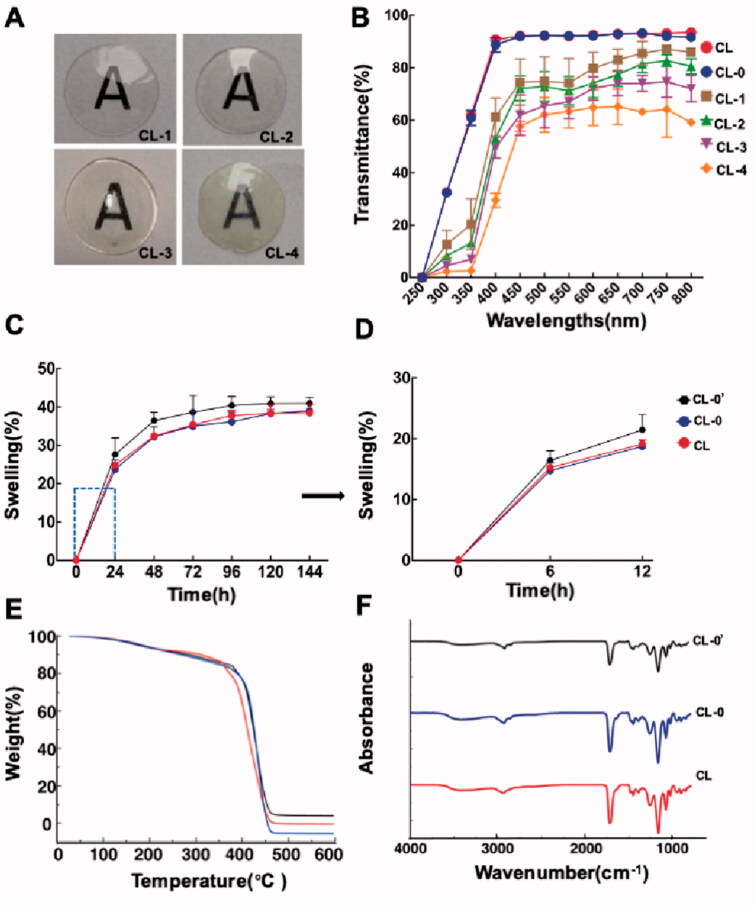
Physical characterizations of contact lenses. (A) Transparency; (B) optical transmittance; (C, D) swelling ratio; (E) thermogravimetric analysis; (F) Fourier transform infrared spectroscopy.

Rutin-encapsulated gelatin hydrogel/contact lens composites were successfully constructed by both *in situ* free radical polymerization and CMC/NHS crosslinking reactions. Compared with the traditional contact lens, the addition of gelatin did not significantly change the transparency (Figure 2(A), Figure S1, supporting information) and the optical transmittance (CL-0 vs. CL, 93.2% ± 4.5% vs. 93.4% ± 1.1%, Figure 2(B)) of contact lenses. Then, the effects of rutin amount on the transparency of the composites were investigated. As shown in Figure 2(A,B), with the increase of rutin amount from 0% to 0.6%, CL-1 and CL-2 were transparent and colorless with optical transmittance of 87.0% ± 0.9% and 82.7% ± 2.2%, respectively. When the rutin amount further increased to 1.0 wt% and 1.3 wt%, the optical transmittances were gradually decreased to 74.7% ± 5.9% (CL-3) and 64.1% ± 10.5% (CL-4), respectively. Therefore, the contact lenses CL-1 and CL-2 can be adopted as ideal rutin carriers with high transparency.

**Figure 3.**
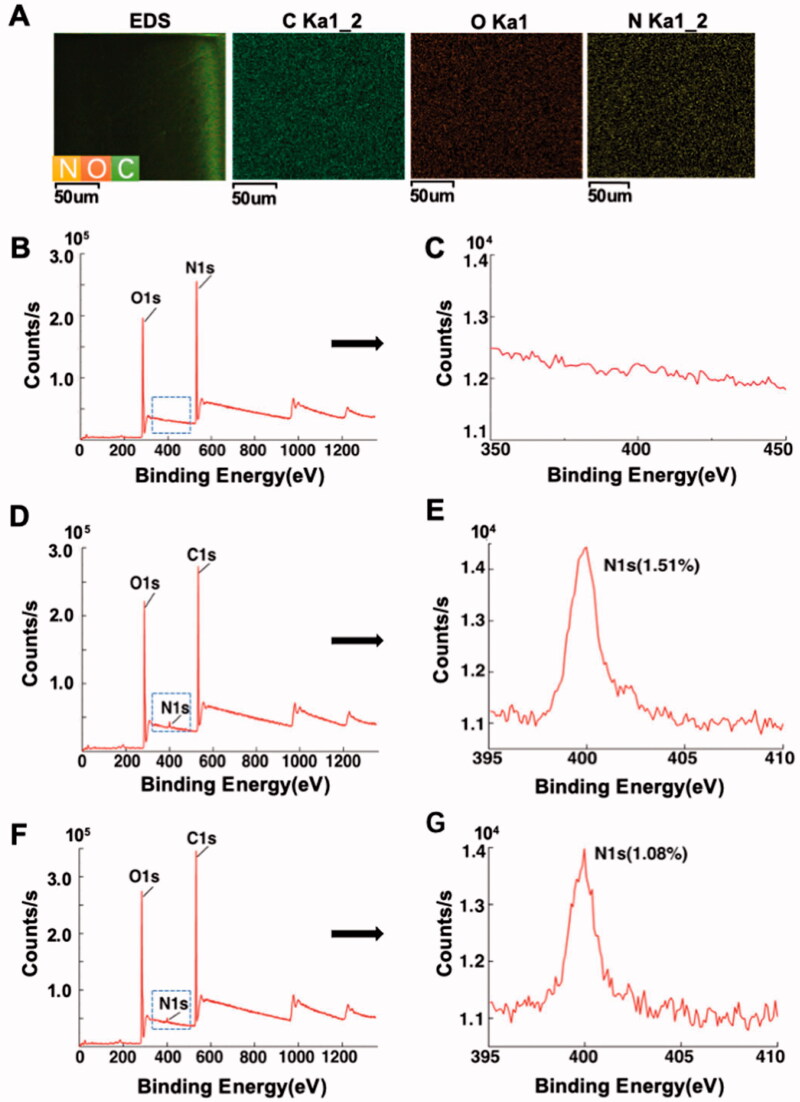
Elemental analysis of the composites. (A) Carbon, oxygen and nitrogen distributions in gelatin hydrogel/contact lens composites; nitrogen content in (B,C) traditional contact lenses, (D,E) gelatin hydrogel/contact lens composites (CL-0), (F,G) gelatin hydrogel/contact lenses without CMC/NHS (CL-0’).

As observed in Figure 2(C,D), after immersion in deionized water for 24 h, CL-0′ (21.4% ± 2.5%) showed a higher swelling rate than CL-0 (18.7% ± 1.1%) and CL (19.1% ± 0.6%) groups. The results showed that the addition of gelatin increased the swelling rate of contact lenses, while the CMC/NHS crosslinking reaction of gelatin further decreased the swelling rate of contact lenses. TGA test confirmed that the material has good thermal stability and thermoplasticity (Figure 2(E)). FTIR analysis revealed no obvious changes in the characteristic peaks between CL-0 and CL. The absent signal of gelatin might be attributed to the low gelatin content in the prepolymeriation solution (Figure 2(F)). EDS analysis demonstrated the even distribution of three elements (C, O, and N) in the composites (Figure 3(A)). The presence of nitrogen element in EDS confirmed the successful introduction of gelatin during free radical polymerization for the preparation of rutin-encapsulated gelatin hydrogel/contact lens composites. XPS analysis reflected that the absence of nitrogen element in CL group, the presence of nitrogen element in CL-0 group and CL-0′ group. Due to the different chemical binding methods, the nitrogen contents in the CL-0 group and CL-0′ group were 1.5% and 1.1%, respectively (Figure 3(B–G)).

### 3.2. *In vitro* rutin release

**Figure 4.**
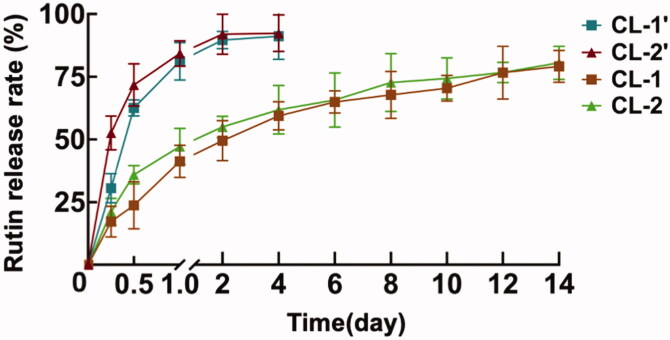
*In vitro* rutin release from rutin-encapsulated gelatin hydrogel/contact lenses composites without (CL-1’, CL-2’) and with CMC/NHS (CL-1, CL-2) crosslinking.

CL-1 and CL-2 were selected for investigating *in vitro* sustained release of rutin due to their good transparency and optic transmittance. The rutin content was detected at different time points for calculating the cumulative release rates of rutin. As Figure 4 reported, the cumulative release rates of rutin from CL-1 and CL-2 were 79.1% and 80.5%, respectively. Moreover, both CL-1and CL-2 could sustainedly release rutin for up to 14 days, which proves a higher sustained-release capacity than that of the composites (CL-1′, CL-2′) with the cumulative rates of 91.2% in 4 days.

### 3.3. *In vitro* biocompatibility and *in vitro* wound healing

**Figure 5.**
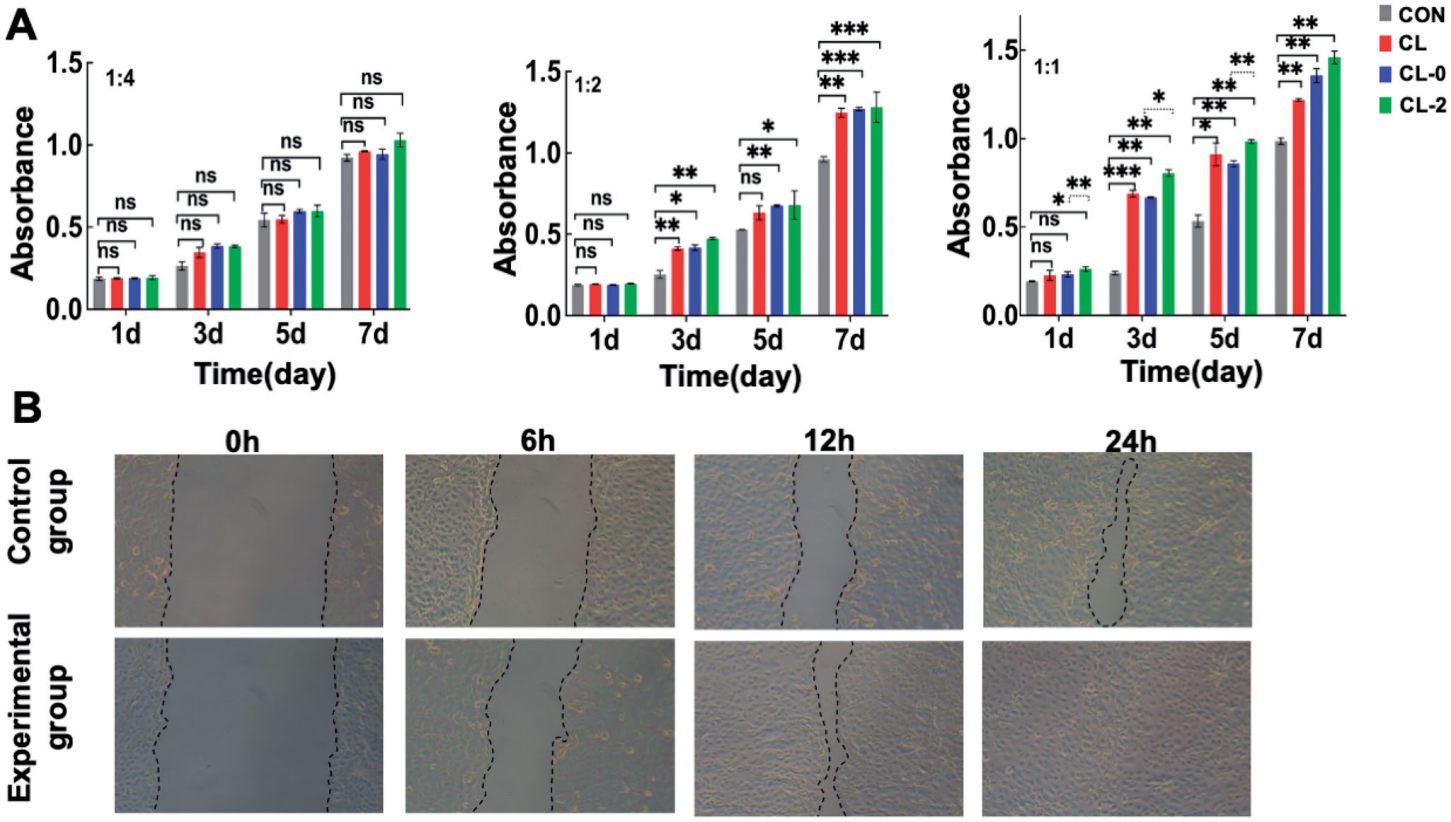
(A) Cytotoxicity, proliferation, and (B) scratch test of the contact lenses on HCECs.

Cytotoxicity and proliferation tests were performed to evaluate the biocompatibility of CL, CL-0, and CL-2. As Figure 5(A) indicated, the viabilities of HCECs in CL-0 and CL-2 groups were similar or superior to those in the negative control and CL groups. The results convey that CL-0 and CL-2 were nontoxic, and had no inhibitory effect on the proliferation of HCECs, which implies satisfactory biocompatibility of the composite. In addition, the scratch damage was repaired faster in the CL-2 group than in the CL group (Figure 5(B)), which suggests a positive effect of rutin-encapsulated gelatin hydrogel/contact lens composites on the damage repair of HCECs.

### 3.4. *In vivo* biocompatibility and *in vivo* wound healing

**Figure 6.**
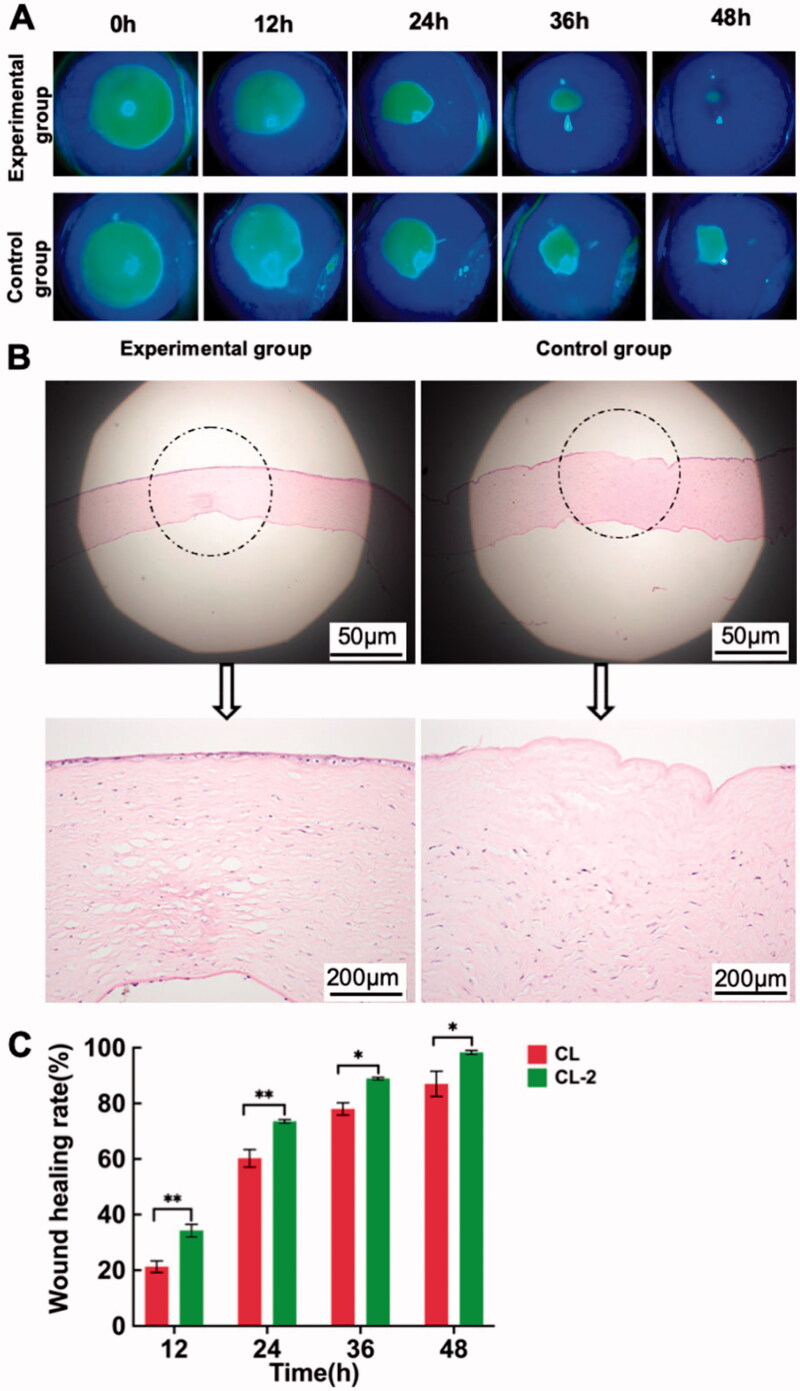
(A) Slit-lamp photographs of rabbit corneas at 12, 24, 36, and 48 h post-operation; (B) hematoxylin and eosin staining of rabbit corneas at 48 h post-operation; (C) wound healing rates of two groups at different post-operation times (*P < .05, **P < .01).

The corneas were observed by slit lamp microscope after CL-2 was continuously worn for 6 h per day in 4 successive days. Results showed that there was no edema or inflammation in corneas, illustrating the good *in vivo* biocompatibility of rutin-encapsulated gelatin hydrogel/contact lens composites (Figure 6(A)). Subsequently, the corneal injury model of rabbits was used to investigate the effect of the composite on corneal wound healing. It was found that corneal wounds were healed faster in the CL-2 group than in the CL-0 group. At 48 h post-operation, the corneal injury was basically cured in the CL-2 group (healing rate, 98.3% ± 0.7%, Figure 6(B)), while the damage was still present in the CL-0 group (healing rate, 87.0% ± 4.5%, Figure 6(C)).

### 3.5. Proteomic profile identification

**Figure 7.**
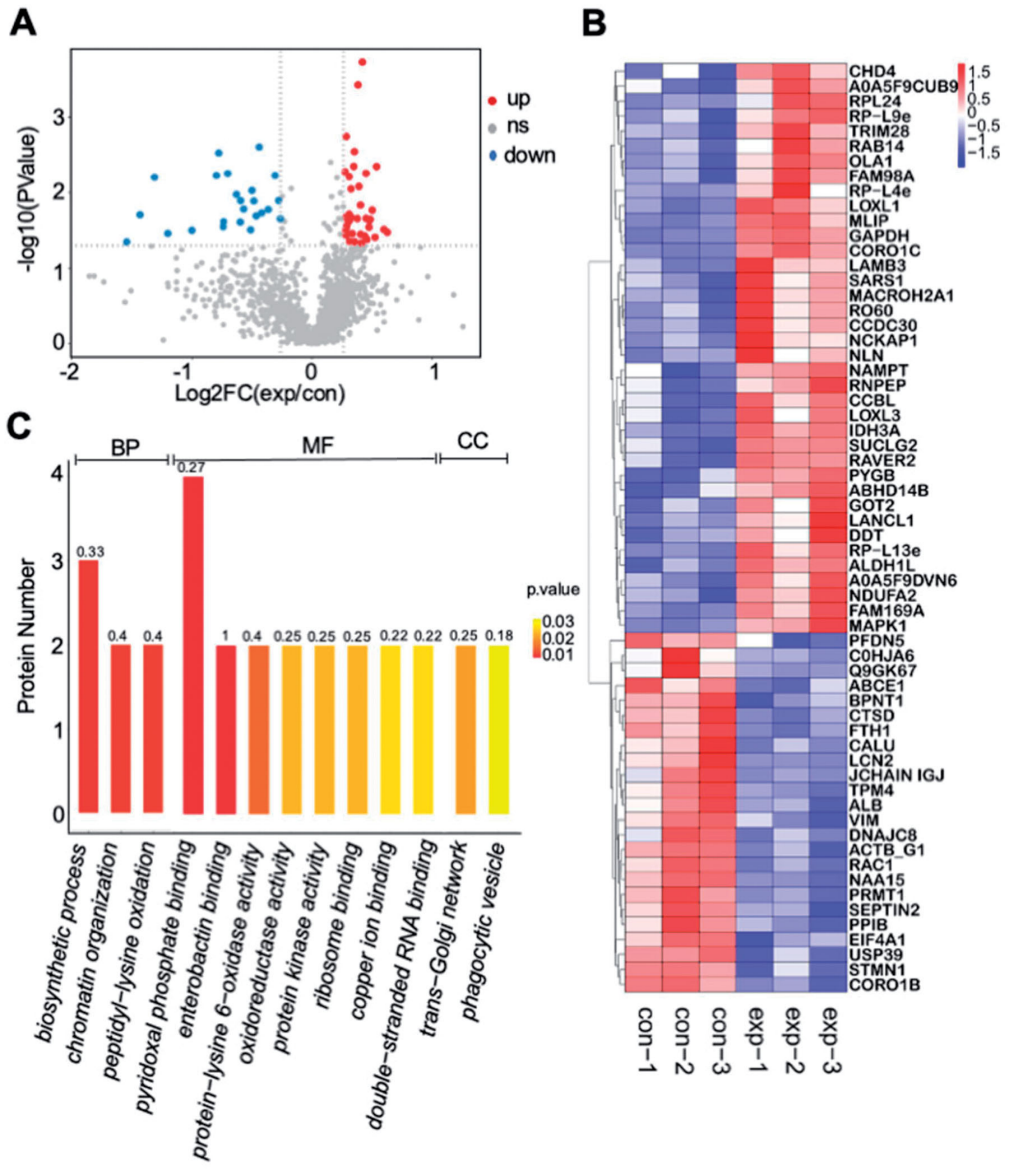
Proteomic analysis of rabbit corneas. (A) Volcano map displaying differential proteins in the experimental group (CL-2) and control group (CL-0); (B) heat map of significantly different proteins with annotated gene names; (C) gene annotation of GO enrichment (top 20) in significantly different proteins with *p* < .05.

A database search of the chromatogram from LC-MS/MS provided the proteins composition of corneas in the control (CL-0) and experimental (CL-2) groups. A total of 1486 proteins were identified from corneal samples. Compared with CL-0 group, 24 proteins were down-regulated and 38 proteins were up-regulated in CL-2 group (FC < 0.83 or FC > 1.2). The differential proteins were presented in the volcano map and the heat map (Figure 7(A,B)). GO enrichment analysis of these proteins (top 20 levels, Figure 7(C)) revealed that the up-regulated proteins were mainly enriched in the positive regulation of nuclear genetic organization and cytoplasmic energy metabolism, while the down-regulated proteins were enriched in the negative regulation of transmembrane transport and double-stranded RNA to promote transcription. In consideration of the molecular function category, the up-regulated proteins were mostly engaged in the biosynthetic process and energy metabolism including the chromatin organization, ribosome binding, and oxidoreductase activity, which was different from the enrichment of down-regulated proteins in double-stranded RNA binding regulator activity and phagocytic vesicle activity (Figure 7(C)).

## 4. Discussion

As a highly regulated biological process, corneal wound healing needs a well-orchestrated and dynamic response such as cell migration, proliferation, and tissue remodeling (Nishida et al., 2015; Zhu et al., 2019; Ziaei et al., 2018). When the injury occurred in cornea epithelial cells, a highly organized cascade of events would happen in three stages, including disruption of hemidesmosomes, the proliferation of migrated cells and remodeling of hemidesmosomes and extracellular matrix (Khosravimelal et al., 2021). Many cytokines and signal pathways are involved in this process, but the mechanism is not clear. In the study, we fabricated rutin-encapsulated biocompatible gelatin hydrogel/contact lens composites to investigate their roles in corneal wound healing, and then explore the potential signaling pathways by proteomics technique.

Hydrogel is an ideal tissue engineering scaffold and medicine material with non-immunogenicity, good biocompatibility, and biodegradability (Al-Kinani et al., 2018; Basu et al., 2018; Delplace et al., 2016; Feng et al., 2016; Griffin et al., 2015; Griffin et al., 2021; Hou et al., 2019; Yang et al., 2021). These properties made hydrogels desirable for cornea wound healing studies. However, the low mechanical strength to some extent limited the wide application of hydrogels (Khosravimelal et al., 2021; Koetting et al., 2015). Traditional contact lenses are important drug-delivery systems for the treatment of ophthalmic diseases (Ciolino et al., 2014; Huang et al., 2016; Kim et al., 2014; Qin et al., 2017; Ross et al., 2019). By combining the merits of hydrogel and contact lenses, it could be interesting to introduce biocompatible gelatin into contact lenses. However, the resulting contact lenses showed an unideal drug-release time (about 4 days). It might be attributed to the physical properties of gelatin hydrogel/contact lens composite, which was synthesized by co-polymerization of HEMA/MAA (70/30, 70 wt%), EGDMA (0.45 wt %) and acrylated gelatin. The insufficient interaction force between the composite and rutin affected the sustained release ability of the composite.

There are residual amino and carboxylic acid after acrylation of gelatin, which indicated that acrylated gelatin can participate in both free radical polymerization and CMC/NHS crosslinking reaction. Thus, the rutin-encapsulated gelatin hydrogel/contact lens composites were prepared by dual crosslinking reaction, exhibiting good biocompatibility and sustained drug-release capacities (14 days). It could be deduced that dual crosslinking reaction altered the physical properties of the composite, and further improved the capacity for sustained release of rutin. Finally, the rutin delivery system was successfully applied in promoting corneal wound healing (healing rate, 98.3% ± 0.7%).

LC-MS/MS basted proteomics technique was used to clarify the potential signaling pathways in corneal wound healing. Finally, 1486 proteins were identified, of which 24 proteins were down-regulated, and 38 proteins were up-regulated compared with the control group. The KEGG and GO enrichment analysis suggested that the composite might contribute to corneal wound healing through the ERK/MAPK and PI3K/AKT signaling pathways. The results were consistent with previous studies regarding the involvement of MAPK and PI3K/Akt signaling pathways in wound healing (Kiang et al., 2020; Liu et al., 2019; Li et al., 2016; Ljubimov & Saghizadeh, 2015; Zhu et al., 2020). Liu et al. considered that the MAPK pathway might take part in the corneal epithelial response to injuries and the tetrahedral framework nucleic acids could promote the corneal epithelial wound healing by upregulating the phosphorylation level of ERK1/2 and p38 (Liu et al., 2019). Zhu et al. found that the secreted protein acidic and rich in cysteine (SPARC) promotes proliferation of limbal epithelial stem cell and corneal wound healing through JNK and p38-MAPK signaling pathways (Zhu et al., 2020). Li et al. corroborated that the MAPK/ERK and PI3K/AKT pathways play a key role in promoting wound surface re-epithelialization, accelerating angiogenesis, and expediting collagen maturity (Li et al., 2016). Besides, Kiang et al. demonstrated that the NF-κB-AKT-MAPK network can reduce combined injury-induced ileum injury by balancing the homeostasis of anti-inflammatory and pro-inflammatory cytokines (Kiang et al., 2020). In the study, GO analysis revealed that the MAPK-1 protein related to the MAPK signaling pathway positively regulated the cytoskeleton remodeling and cell proliferation, while the Rac1 protein related to the PI3K/AKT signaling pathway negatively regulated the apoptosis and inflammatory response. The combined use of rutin-encapsulated gelatin hydrogel/contact lens composites and proteomics presented the comprehensive proteome atlas and indicated the ERK/MAPK and PI3K/AKT signaling pathways in corneal wound healing.

## 5. Conclusions

In the work, a biocompatible rutin-encapsulated gelatin hydrogel/contact lens composite was successfully prepared by dual crosslinking reactions including *in situ* free radical polymerization and CMC/NHS crosslinking reaction. The composite, as a rutin delivery system, can sustainedly release rutin for up to 14 days and promote the wound healing of rabbit corneas in 2 days. The proteomics analysis certified that the corresponding molecular mechanism might be related to ERK/MAPK and PI3K/Akt signaling pathways. Further efforts should be used to extensively explore the clinical applications of the therapeutic composites for corneal wound healing in humans. It would also be interesting for researchers to expand applications of the composites to treat other human diseases with the rich biological activities of rutin.

## Supplementary Material

Supplemental Material
