# Supplementary material for: Gelatin hydrogel/contact lens composites as rutin delivery systems for promoting corneal wound healing
Source: Drug Deliv. 2021 Sep 25;28(1):1951–61. doi: 10.1080/10717544.2021.1979126 (PMC8475096; doi:10.1080/10717544.2021.1979126)

Supporting information

Construction of gelatin hydrogel/contact lens composites as rutin delivery systems for promoting corneal wound healing

Lianghui Zhao ^1,2^, Xia Qi ^1,2^, Tao Cai ^1,2^, Zheng Fan ^1,2^, Hongwei Wang ^1,2*^, Xianli Du ^1,2*^

^1^Qingdao Eye Hospital of Shandong First Medical University, Qingdao, Shandong 266071, China;

^2^State Key Laboratory Cultivation Base, Shandong Provincial Key Laboratory of Ophthalmology, Shandong Eye Institute, Shandong First Medical University & Shandong Academy of Medical Sciences, Qingdao, Shandong 266071, China.

***** Correspondence authors:

Hongwei Wang (E-mail: whw20051256@163.com, Tel.: +86-532-85899332);

Xianli Du (E-mail: lilibestever@126.com, Tel.: +86-532-85881625).

Gradient conditions for EASY-nLC 1200 nano liquid chromatography.

Table S1

| Time [mm:ss] | Duration [mm:ss] | Flow [nL/min] | Mixture [%B] |
| --- | --- | --- | --- |
| 00:00 | 00:00 | 600 | 5 |
| 03:00 | 03:00 | 600 | 6 |
| 42:00 | 39:00 | 600 | 18 |
| 53:00 | 14:00 | 600 | 27 |
| 54:00 | 01:00 | 600 | 95 |
| 60:00 | 06:00 | 600 | 95 |

Table S2

| Time [mm:ss] | Duration [mm:ss] | Flow [nL/min] | Mixture [%B] |
| --- | --- | --- | --- |
| 00:00 | 00:00 | 600 | 5 |
| 03:00 | 03:00 | 600 | 7 |
| 42:00 | 39:00 | 600 | 19 |
| 53:00 | 14:00 | 600 | 27 |
| 54:00 | 01:00 | 600 | 95 |
| 60:00 | 06:00 | 600 | 95 |

Table S3

| Time [mm:ss] | Duration [mm:ss] | Flow [nL/min] | Mixture [%B] |
| --- | --- | --- | --- |
| 00:00 | 00:00 | 600 | 6 |
| 03:00 | 03:00 | 600 | 7 |
| 42:00 | 39:00 | 600 | 21 |
| 53:00 | 14:00 | 600 | 29 |
| 54:00 | 01:00 | 600 | 95 |
| 60:00 | 06:00 | 600 | 95 |

Table S4

| Time [mm:ss] | Duration [mm:ss] | Flow [nL/min] | Mixture [%B] |
| --- | --- | --- | --- |
| 00:00 | 00:00 | 600 | 8 |
| 03:00 | 03:00 | 600 | 11 |
| 42:00 | 39:00 | 600 | 22 |
| 53:00 | 14:00 | 600 | 30 |
| 54:00 | 01:00 | 600 | 95 |
| 60:00 | 06:00 | 600 | 95 |

Table S5

| Time [mm:ss] | Duration [mm:ss] | Flow [nL/min] | Mixture [%B] |
| --- | --- | --- | --- |
| 00:00 | 00:00 | 600 | 9 |
| 03:00 | 03:00 | 600 | 11 |
| 42:00 | 39:00 | 600 | 24 |
| 53:00 | 14:00 | 600 | 31 |
| 54:00 | 01:00 | 600 | 95 |
| 60:00 | 06:00 | 600 | 95 |

Table S6

| Time [mm:ss] | Duration [mm:ss] | Flow [nL/min] | Mixture [%B] |
| --- | --- | --- | --- |
| 00:00 | 00:00 | 600 | 12 |
| 03:00 | 03:00 | 600 | 15 |
| 42:00 | 39:00 | 600 | 26 |
| 53:00 | 14:00 | 600 | 34 |
| 54:00 | 01:00 | 600 | 95 |
| 60:00 | 06:00 | 600 | 95 |

**HPLC conditions**: chromatographic column, 150 mm ×4.6 mm ID, 5.0 μm C18 particles; injection volume, 5 μL; flow rate, 1.0 mL/min; column temperature, 40 ℃; elution condition, 60% ACN/H_2_O (v/v) solution for 10.0 min.

**Figure S1**


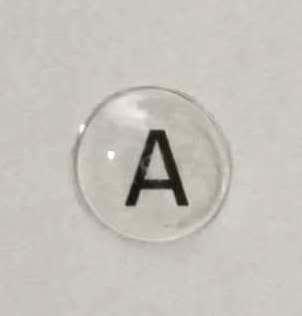

Supplement: Supplemental Material [file IDRD_A_1979126_SM0369.docx]
